# Multiple hepatic pseudotumors: A rare presentation of hepatic steatosis

**DOI:** 10.1002/jgf2.412

**Published:** 2020-12-19

**Authors:** Hirohisa Fujikawa, Tsubasa Fukuoka

**Affiliations:** ^1^ Department of Medical Education Studies International Research Center for Medical Education Graduate School of Medicine The University of Tokyo Bunkyo‐ku Japan; ^2^ Department of Internal Medicine Suwa Central Hospital Chino Japan

**Keywords:** diagnostic reasoning, family medicine, gastrointestinal medicine, hospital general medicine, internal medicine

## Abstract

A 39‐year‐old woman was suspected malignancy on computed tomography. The patient was finally diagnosed with multifocal nodular steatosis due to massive alcohol consumption. It is significant to consider hepatic steatosis as a possible differential diagnosis of multiple hypodense hepatic lesions and to take a careful history, which may lead to avoid unnecessary biopsies.

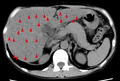

A previously healthy 39‐year‐old woman presented to the hospital with a 1‐day history of general malaise. She drank countless bottles of distilled spirits every day. She had no recent medication history. On examination, she had no abdominal tenderness and no masses palpated. Laboratory tests showed abnormal liver profiles (aspartate aminotransferase 292 U/L, alanine aminotransferase 127 U/L, lactate dehydrogenase 430 U/L, alkaline phosphatase 546 U/L, gamma‐glutamyl transpeptidase 2075 U/L, and total bilirubin 1.24 mg/dL). Hepatitis B virus (HBV) surface antigen, hepatitis C virus (HCV) antibody, antinuclear antibody, and antimitochondrial M2 antibody were all negative. Abdominal ultrasound revealed hepato‐renal echo contrast, liver brightness, and multiple low echoic masses. Abdominal computed tomography depicted multiple hypodense hepatic lesions (Figure 1). The differential diagnoses included metastases, lymphomas, hemangiomatoses, biliary hamartomas, and hepatic steatosis. After 10 days of abstinence from alcohol, malaise and multiple hepatic lesions resolved (Figure 2). Liver function also recovered. Considering her history of massive alcohol consumption, rapid recovery of the hepatic lesions after cutting out alcohol, and negative results of serological markers (HBV, HCV, and autoimmune liver diseases), we diagnosed multifocal nodular steatosis because of alcohol abuse.

**FIGURE 1:**
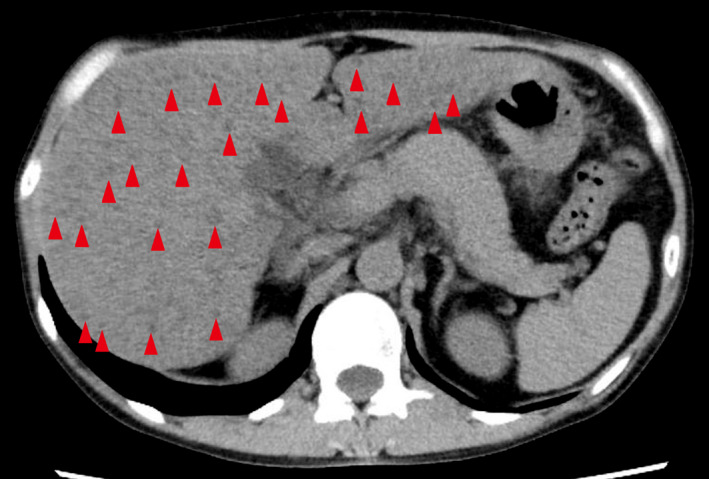
Abdominal computed tomography showing multiple hypodense liver masses

**FIGURE 2:**
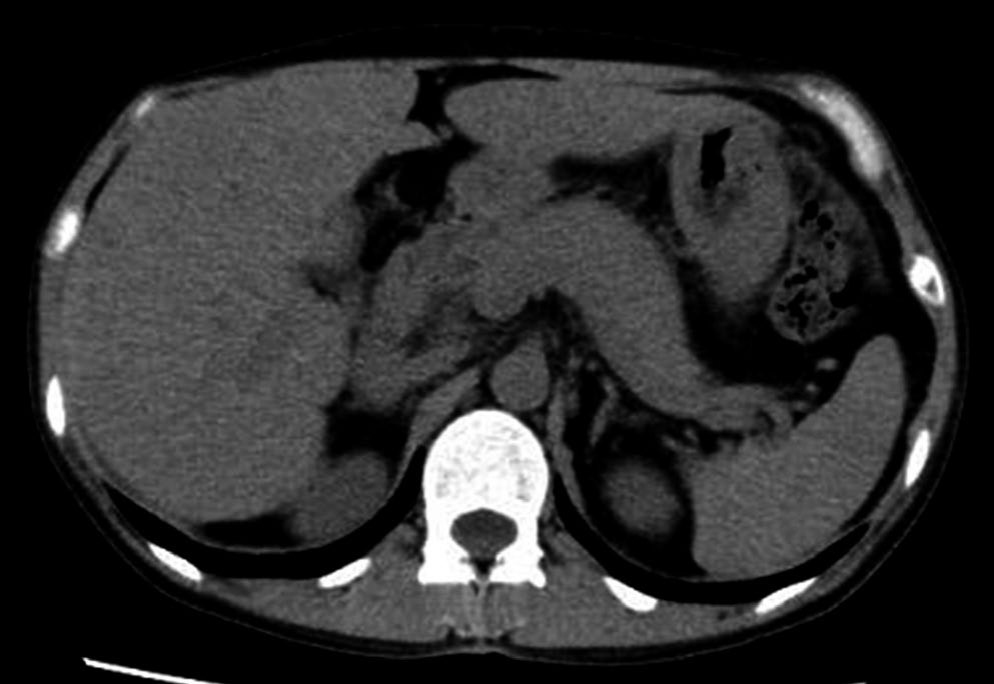
Improvement of multiple hypodense hepatic lesions after ten days of abstinence from alcohol

Hepatic steatosis is a benign and common condition with a prevalence of approximately 10% in general population.^1^ It is characterized by fat deposits in liver parenchyma. It is classified as idiopathic or secondary. Secondary liver steatosis occurs because of underlying conditions such as alcohol use, diabetes, hepatitis, obesity, parenteral nutrition, starvation, and steroid therapy.^2^

Alcohol steatosis is the earliest stage of alcohol‐related liver disease and is usually fully reversible. Although it is commonly asymptomatic, it may present with mild nonspecific symptoms as the present case.^3^ Because it can completely recover within 2 weeks of abstinence, discontinuation of alcohol consumption is essential for treatment.^4^

Although multiple nodular patterns of hepatic steatosis on imaging studies are rare, they may mimic neoplastic conditions (so‐called “pseudotumors”) and require extreme caution.^5^ Because the differential diagnosis of multiple hepatic hypodense masses can be difficult based on imaging alone, a biopsy is frequently performed, especially for excluding malignancy.^6^ However, in some cases, biopsy can be avoided. According to previous case reports, contrast‐enhanced ultrasound and magnetic resonance imaging may be useful to avoid misdiagnosis and unnecessary biopsies of hepatic tumors.^5^, ^7^ In the present case, careful history taking of alcohol consumption was an important clue to diagnosis.

## CONFLICT OF INTEREST

The authors have stated explicitly that there are no conflicts of interest in connection with this article.

## INFORMED CONSENT

Informed consent was obtained from the patient to publish the image.
